# Front and Back Movement Analysis of a Triangle-Structured Three-Wheeled Omnidirectional Mobile Robot by Varying the Angles between Two Selected Wheels

**DOI:** 10.1155/2016/7612945

**Published:** 2016-02-15

**Authors:** A. P. Mohanraj, A. Elango, Mutra Chanakya Reddy

**Affiliations:** ^1^School of Mechanical Engineering, SASTRA University, Thanjavur, Tamil Nadu 613401, India; ^2^Department of Mechanical Engineering, ACCET, Karaikudi, Tamil Nadu 630004, India

## Abstract

Omnidirectional robots can move in all directions without steering their wheels and it can rotate clockwise and counterclockwise with reference to their axis. In this paper, we focused only on front and back movement, to analyse the square- and triangle-structured omnidirectional robot movements. An omnidirectional mobile robot shows different performances with the different number of wheels and the omnidirectional mobile robot's chassis design. Research is going on in this field to improve the accurate movement capability of omnidirectional mobile robots. This paper presents a design of a unique device of Angle Variable Chassis (AVC) for linear movement analysis of a three-wheeled omnidirectional mobile robot (TWOMR), at various angles (*θ*) between the wheels. Basic mobility algorithm is developed by varying the angles between the two selected omnidirectional wheels in TWOMR. The experiment is carried out by varying the angles (*θ* = 30°, 45°, 60°, 90°, and 120°) between the two selected omniwheels and analysing the movement of TWOMR in forward direction and reverse direction on a smooth cement surface. Respectively, it is compared to itself for various angles (*θ*), to get its advantages and weaknesses. The conclusion of the paper provides effective movement of TWOMR at a particular angle (*θ*) and also the application of TWOMR in different situations.

## 1. Introduction

Omnidirectional mobile robots are useful in a large variety of indoor service applications such as industrial, medical, and domestic purposes. Omnidirectional mobile robots provide high mobility compared to the more common car-like robots. Some of the omnidirectional mobile robot platforms are designed for use in the highway maintenance and congested environment [1]. The omnidirectional chassis has many forms; the three-wheel and four-wheel schemes are the most familiar. The number of wheels and the layout can both affect the performances of the chassis [2–4]. There have been several works on the applications of using omnidirectional mobile robots [5, 6], the development of three special wheeled structures, and also the isotropy analysis of the mobile robots and kinematic analysis. There have been several works on the impact of the number of wheels and layouts to the performances of omnidirectional chassis [7–9]. There are studies on motion planning algorithms of an omnidirectional mobile robot, which helps us to understand the movement of an omnidirectional mobile robot [10–12]. Caster drive mechanism for the omnidirectional mobile robot platforms is also attractive aspect [13–20].

The angle variations between those two wheels will enable us to analyse the sideways movement of triangular structure and crossway movements of square structure omnidirectional robot. In this paper, the forward and reverse movement analysis and deflections caused due to the various angles between two wheels, like *θ* = 30°, 45°, 60°, 90°, and 120°, were experimentally analysed. Therefore, the paper attempts to analyse how the angle variations between the two wheels are giving impact on the movement of omnidirectional mobile robots. The theoretical analysis and experimental tests were conducted in order to demonstrate the performance of TWOMR.

## 2. The Omnidirectional Mobile Robot Platform

**Figure 1:**
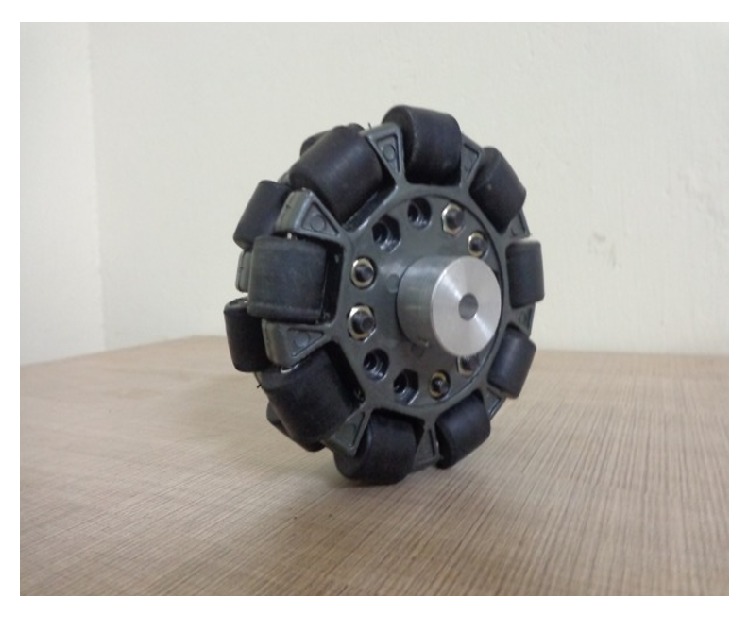
Omnidirectional wheel.

The double row omnidirectional wheel is shown in Figure 1, and specifications of omniwheel and DC motor are mentioned in Tables 1 and 2, respectively. It can move forward, backward, and sideways also by using the rotation of the rollers. The Pro-E design and the prototype of three-wheeled omnidirectional mobile robot (TWOMR) are shown in Figures 3 and 4.

**Figure 2:**
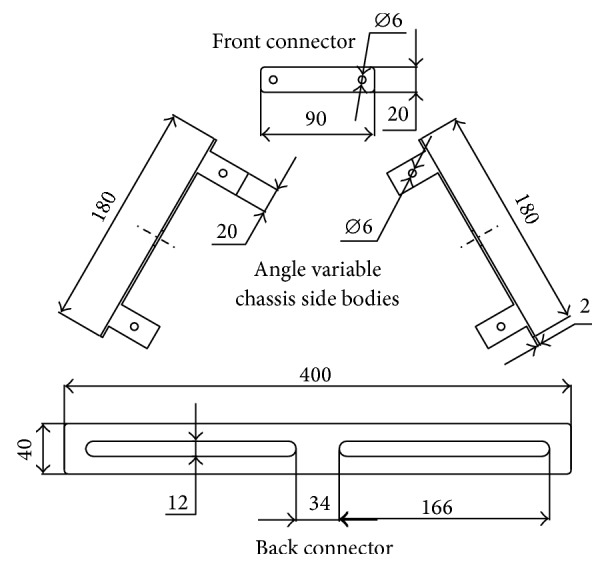
Angle Variable Chassis (AVC) side body and front and back connectors with dimensions (all dimensions are in mm).

To analyse different angles of TWOMR, a unique device, Angle Variable Chassis (AVC), is designed as shown in Figure 2; it is having two “side bodies,” having 180 mm length for a 100 mm diameter omniwheel. Two side extensions are there in each “side body” with 6 mm diameter hole. The front connector is designed as 90 mm in length and 20 mm in width. It is having 2 holes in 6 mm diameter. The back connector is having a length of 400 mm and width of 40 mm.

A 12 mm slot has been created for nearly 160 mm long, to connect with the back side of the body. All the structures had been fabricated in 2 mm thick mild steel plate, for its stiffness. “Side body” and front and back connector plates were connected by bolt, nut, and some washers. By adjusting the “chassis side bodies” of the “Angle Variable Chassis” (AVC), the angle between the wheels can be varied.

**Figure 3:**
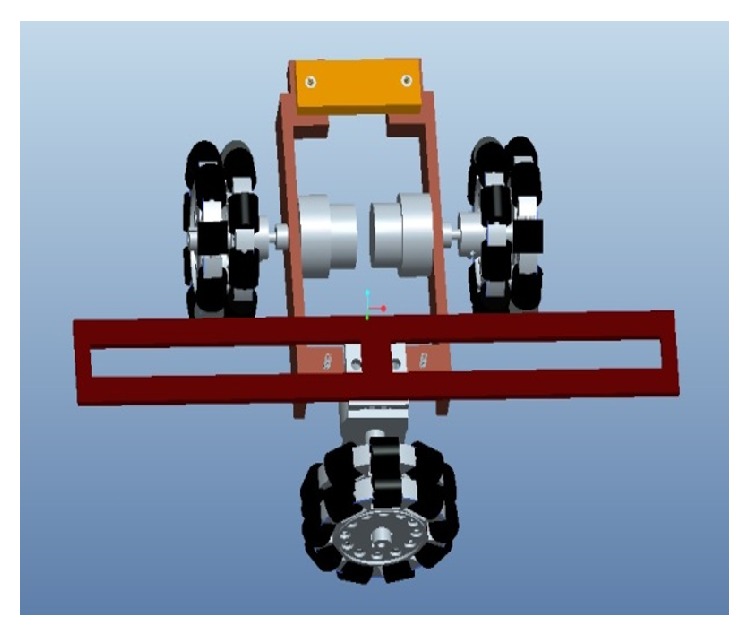
Pro-E design of TWOMR.

**Figure 4:**
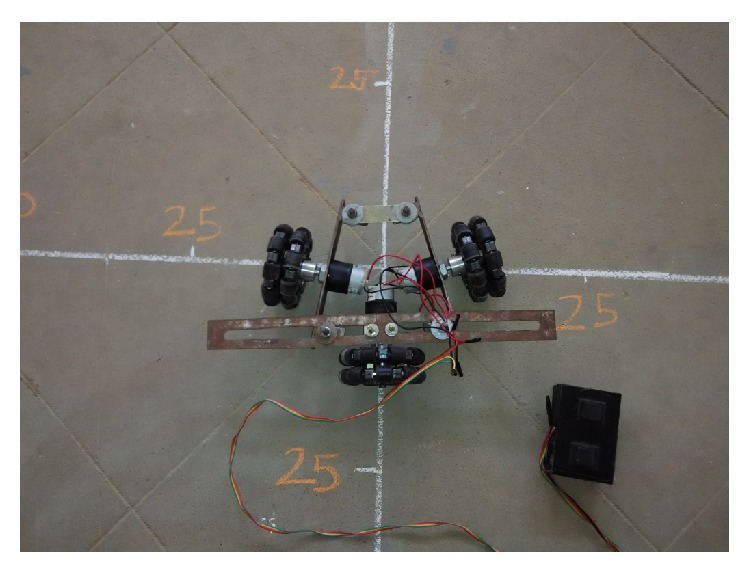
Prototype of TWOMR.

The proposed Pro-E design and prototype of TWOMR are shown in Figures 3 and 4, respectively. The three-wheeled omnidirectional mobile robots are capable of achieving 3 DOF motions by driving three independent actuators. So the mobile robot used in this research is a holonomic omnidirectional mobile robot. The chassis is designed in such a way that the angles between omniwheel 1 and omniwheel 2 from Figure 4 can be maintained from 10° to 170°.

**Figure 5:**
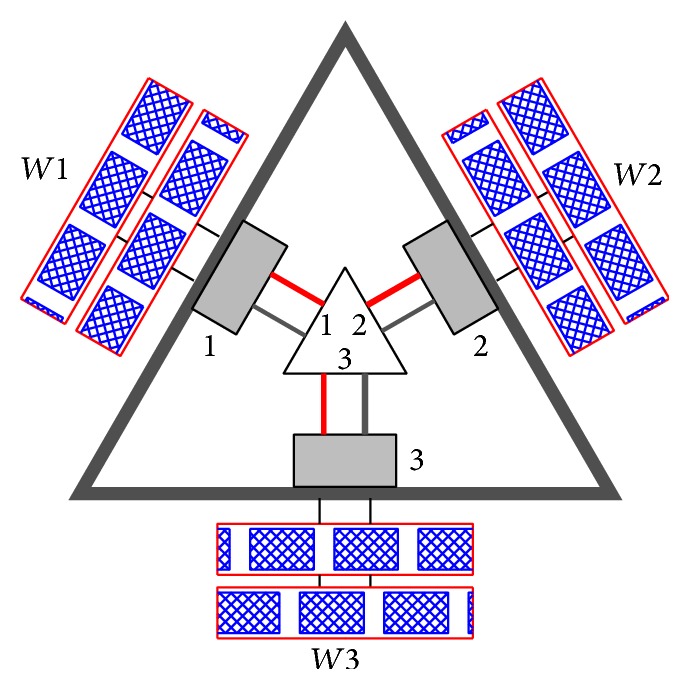
The circuit diagram of TWOMR (1, 2, and 3 are DC motors and *W*1, *W*2, and *W*3 are omniwheels).

Hence, the experiments are carried out by varying the angles between omniwheel 1 and omniwheel 2, neglecting omniwheel 3. Each wheel is fixed with an independent DC motor actuator and each DC motor actuator is controlled by a remote controller. The circuit diagram is shown in Figure 5. Only omniwheel 1 and omniwheel 2 are used for the movement of the mobile robot; omniwheel 3 is used to provide the robot stability but not for the movement of the mobile robot. Omniwheels 1 and 2 should move with the same speed and each omniwheel has the capability to drive independently. Each omniwheel driven by a DC motor consists of eighteen orthogonal rollers and provides two-directional movements. With a proper mechanical design of chassis, the TWOMR can be able to move in forward and reverse direction by varying the angles between omniwheel 1 and omniwheel 2 from *θ* = 10° to 170°.

This Angle Variable Chassis (AVC) is designed to vary the angles of triangle-structured omnidirectional mobile robot which is having 60° in all sides (equilateral triangle). The experiments were conducted for 10° to 170°; in this paper, the experimental tests are shown for some important angles used in a triangle, like 30°, 45°, 60°, 90°, and 120°, because when the angle is 0°, omniwheel 1 and omniwheel 2 are parallel to each other. The omniwheels almost acted like a normal wheel. When the angles were increased from 0°, then two omniwheels were creating resultant force.

For angles between 0° and 30°, the TWOMR did not show much deviation. When it is reached at the range of 30°, it has shown some deviations. When the angles are beyond 120° to 180°, omniwheel 1 and omniwheel 2 could not create the desired resultant force for its movement. When the angles have been increased beyond 120° to 180°, the two omniwheels are creating almost equal and opposite forces, so that the TWOMR is struggling to create the front and back movements. So, in this paper, the experimental analysis has been shown up to 120°.

## 3. Kinematic Model of the TWOMR

**Figure 6:**
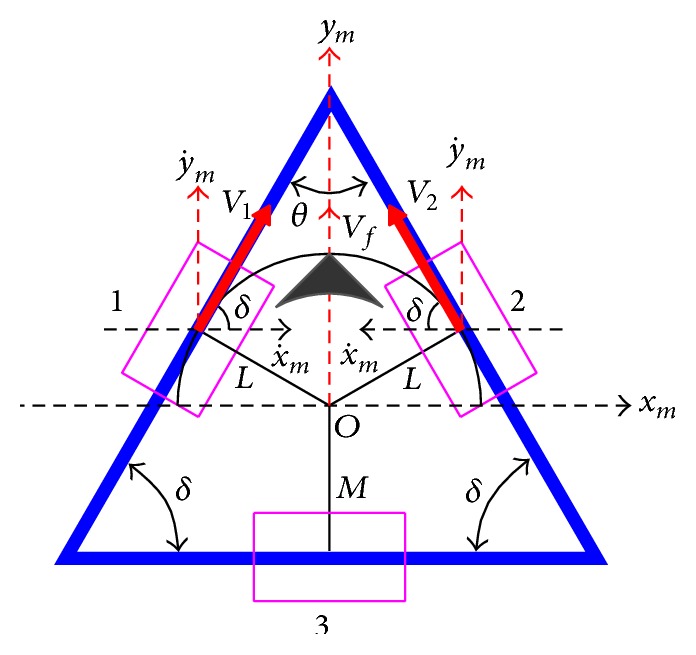
Forward movement schematic of chassis.

**Figure 7:**
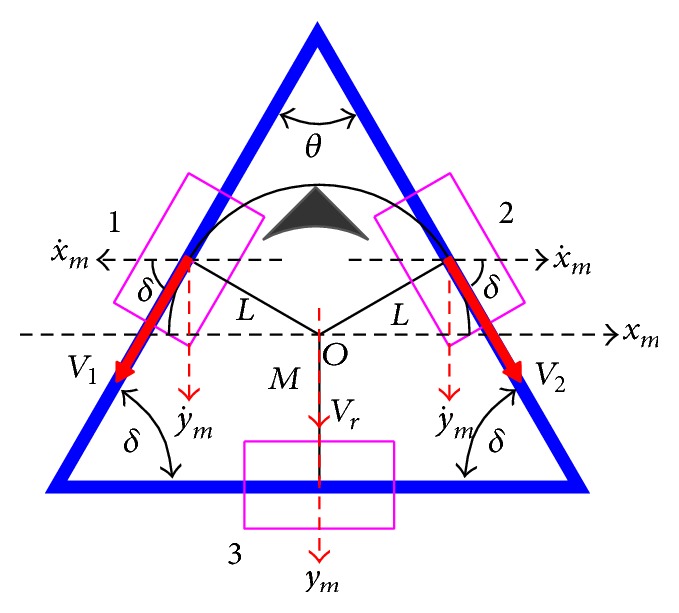
Reverse movement schematic of chassis.

The schematic of the chassis is shown in Figures 6 and 7, where a full “black” triangle indicates the forehead of the robot. The length between omniwheels 1 and 2 and centre *O* is *L* and the length between omniwheel 3 and centre *O* is *M*.

The lengths *L* and *M* change according to angle *θ* between omniwheel 1 and omniwheel 2. We define the fact that velocity *V*_1_ and velocity *V*_2_ of omniwheel 1 and omniwheel 2, respectively, are positive when the robot rotates counterclockwise. As shown in Figures 6 and 7, the mobile coordinate frame [*x*_*m*_, *y*_*m*_] is located at the centre of gravity of the robot. The two front wheels (1 and 2) are offset from “*x*_*m*_” by fixed angle *δ*. Angle *δ* changes according to angle *θ* between omniwheel 1 and omniwheel 2.

According to the schematic of the chassis with three omniwheels, we can derive the kinematics model of the TWOMR using trigonometry and geometry. Assume the radius of each omniwheel is *R* and its rotating rate is *ω*_*i*_. We can derive the velocity of each omniwheel by

(1) $$\begin{array}{c} V_{i}=R\omega_{i},\;i=1,2, \end{array}$$

where *i* denotes the *i*th omniwheel. When the TWOMR moves in the forward direction at velocity *V*_*f*_, velocities *V*_1_ and *V*_2_ can be derived as follows:

(2) V1=Rω1=+cos⁡δx˙+sin⁡δy˙,V2=Rω2=−cos⁡δx˙+sin⁡δy˙,

where $\dot{x}$ and $\dot{y}$ denote the velocity of the direction of *x*_*m*_ and *y*_*m*_, respectively. Equations (2) can be rewritten as

(3) $$\begin{array}{c} \left[\begin{array}{c} V_{1}\\ V_{2} \end{array}\right]=\left[\begin{array}{c} R\omega_{1}\\ R\omega_{2} \end{array}\right]=\left[\begin{array}{cc} \cos\left(\delta \right) & \sin\left(\delta \right)\\ -\cos\left(\delta \right) & \sin\left(\delta \right) \end{array}\right]\left[\begin{array}{c} \dot{x}\\ \dot{y} \end{array}\right]. \end{array}$$

The forward resultant velocity (*V*_*f*_) can be written as

(4) $$\begin{array}{c} V_{f}=V_{1}+V_{2}=2\sin\left(\;\delta \;\right)\dot{y}. \end{array}$$

Similarly, when the TWOMR moves in the reverse direction at velocity *V*_*r*_, velocities *V*_1_ and *V*_2_ can be derived as follows:

(5) V1=Rω1=−cos⁡δx˙−sin⁡δy˙,V2=Rω2=+cos⁡δx˙−sin⁡δy˙,

where $\dot{x}$ and $\dot{y}$ denote the velocity of the direction of *x*_*m*_ and *y*_*m*_, respectively.

Equations (5) can be rewritten as

(6) $$\begin{array}{c} \left[\begin{array}{c} V_{1}\\ V_{2} \end{array}\right]=\left[\begin{array}{c} R\omega_{1}\\ R\omega_{2} \end{array}\right]=\left[\begin{array}{cc} -\cos\left(\delta \right) & -\sin\left(\delta \right)\\ \cos\left(\delta \right) & -\sin\left(\delta \right) \end{array}\right]\left[\begin{array}{c} \dot{x}\\ \dot{y} \end{array}\right]. \end{array}$$

The reverse resultant velocity (*V*_*r*_) can be written as

(7) $$\begin{array}{c} V_{r}=V_{1}+V_{2}=-2\sin\left(\;\delta \;\right)\dot{y}, \end{array}$$

where negative sign indicates reverse direction.

## 4. Theoretical Analysis of TWOMR

In the theoretical analysis, some assumptions are made; for example, there are no effect of friction on TWOMR, no effect of omniwheel rollers on omniwheels, and no errors in the design and manufacturing process.

From Figures 6 and 7, the schematic of the chassis and from (4) and (7), the resultant velocity forward and reverse respectively; Table 3 shows the relations.

Table 3 clearly shows that there is an impact of angle (*θ*) on the resultant velocity in both forward (*V*_*f*_) and reverse (*V*_*r*_) direction. As we can see, increasing the angle (*θ*) from 30° to 120°, there is a decrease in the angle (*δ*) from 75° to 30°; the sine value decreases from 75° to 30°. Hence, at an angle (*θ* = 30°), the sine value at 75° is maximum, so maximum velocity is obtained in both forward and reverse direction.

At angle (*θ* = 120°), the sine value at 30° is minimum, so minimum velocity is obtained in both forward and reverse direction. As a result, if the angle (*θ*) between omniwheel 1 and omniwheel 2 increases, there will be a decrease in the resultant velocity in both forward (*V*_*f*_) and reverse (*V*_*r*_) direction and vice versa.

Theoretically, there will not be any deflections or deviations occurring at angles (*θ* = 30°, 45°, 60°, 90°, and 120°) in both forward and reverse direction. The omnidirectional mobile robot has to travel in straight paths in both forward and reverse direction.

Figures 6 and 7 show the forward and reverse resultants, respectively, which are straight paths, and omnidirectional mobile robot is supposed to travel in straight paths without any small deflections or deviations in both forward and reverse direction at all angles (*θ* = 30°, 45°, 60°, 90°, and 120°) theoretically.

The theoretical linear movement analysis for TWOMR at all angles *θ* = 30°, 45°, 60°, 90°, and 120° is shown in Figures 11, 12, 13, 14, and 15 for forward (*V*_*f*_) direction and the theoretical linear movement analysis for the reverse (*V*_*r*_) direction is shown in Figures 17, 18, 19, 20, and 21. The path followed by TWOMR theoretically is termed as the “ideal path” in this paper.

## 5. Experimental Tests

**Figure 8:**
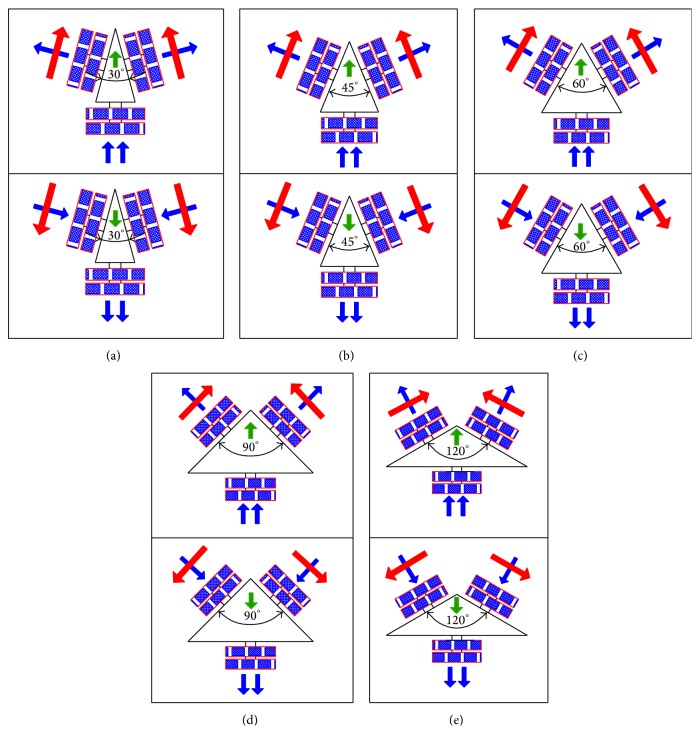
Front and back movement of TWOMR, wheel and roller rotations, when (a) *θ* = 30°, (b) *θ* = 45°, (c) *θ* = 60°, (d) *θ* = 90°, and (e) *θ* = 120°.

Experimental tests are conducted at each angle (*θ* = 30°, 45°, 60°, 90°, and 120°) in forward and reverse direction. The experimental result shows that the TWOMR is deflecting or deviating from its “ideal path.” These deflections are noted and plotted in graphs. Figures 8(a), 8(b), 8(c), 8(d), and 8(e) show the line diagram of the front and back movement analysis at 30°, 45°, 60°, 90°, and 120°.

**Figure 9:**
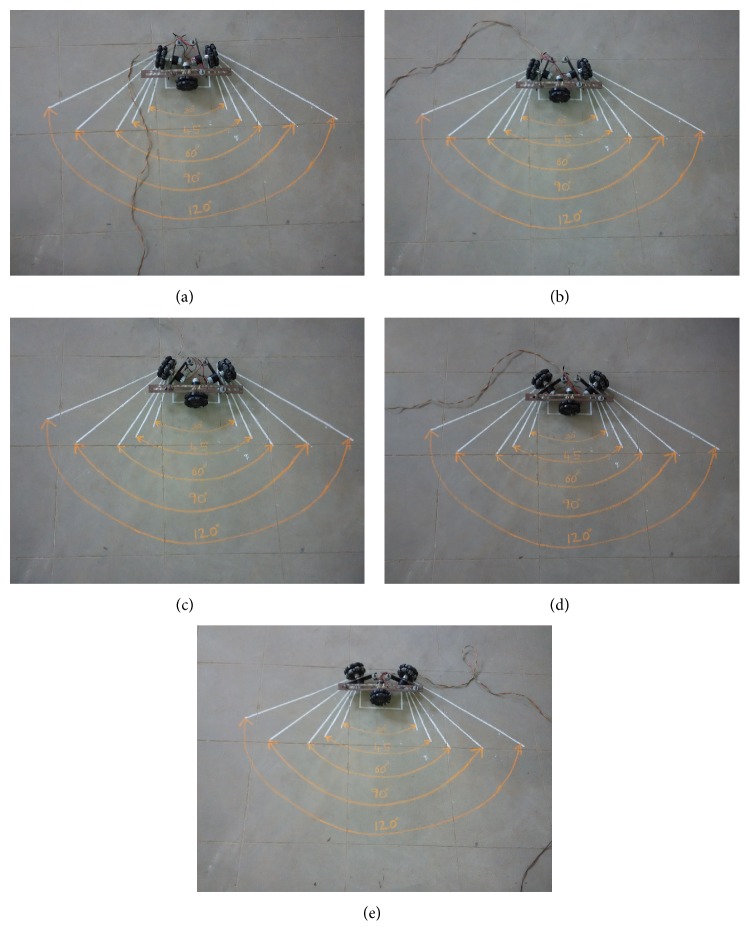
Three-wheeled omnidirectional mobile robot (TWOMR). (a) *θ* = 30°, (b) *θ* = 45°, (c) *θ* = 60°, (d) *θ* = 90°, and (e) *θ* = 120°.

Figures 9(a), 9(b), 9(c), 9(d), and 9(e) show the real-time pictures of the angle between the wheels at 30°, 45°, 60°, 90°, and 120°.

### 5.1. Forward Movement Analysis of TWOMR

For the forward movement analysis of TWOMR, two trials are conducted for each angle (*θ* = 30°, 45°, 60°, 90°, and 120°) and deflection readings are plotted in Figures 11, 12, 13, 14, and 15, respectively.

**Figure 10:**
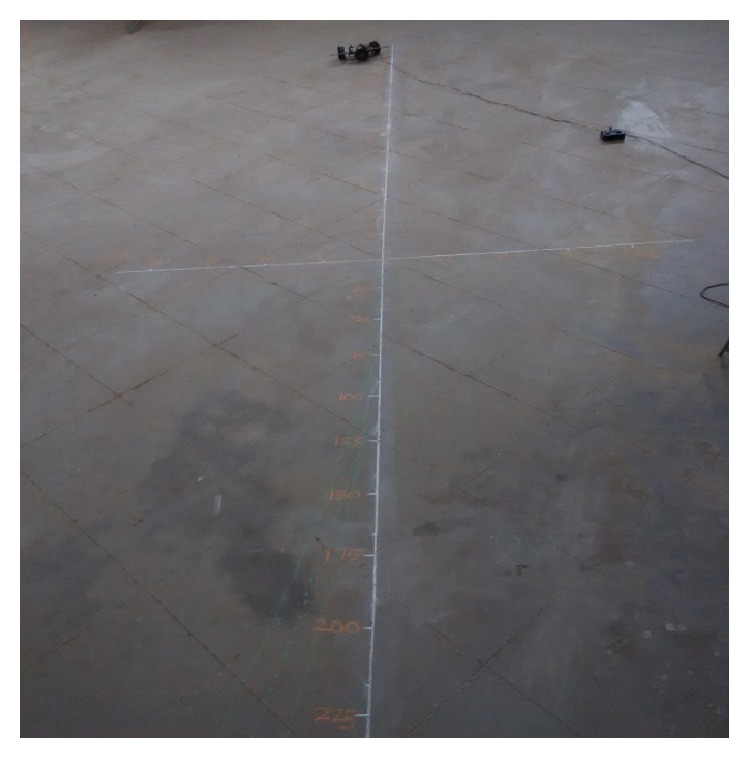
Real-time experiment of forward movement analysis of TWOMR at *θ* = 45°.

**Figure 11:**
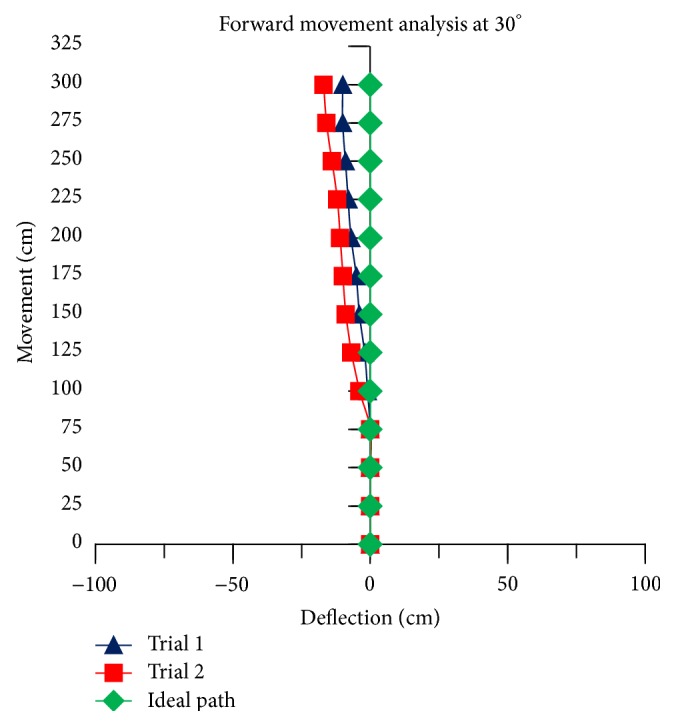
Forward movement versus deflections at *θ* = 30°.

**Figure 12:**
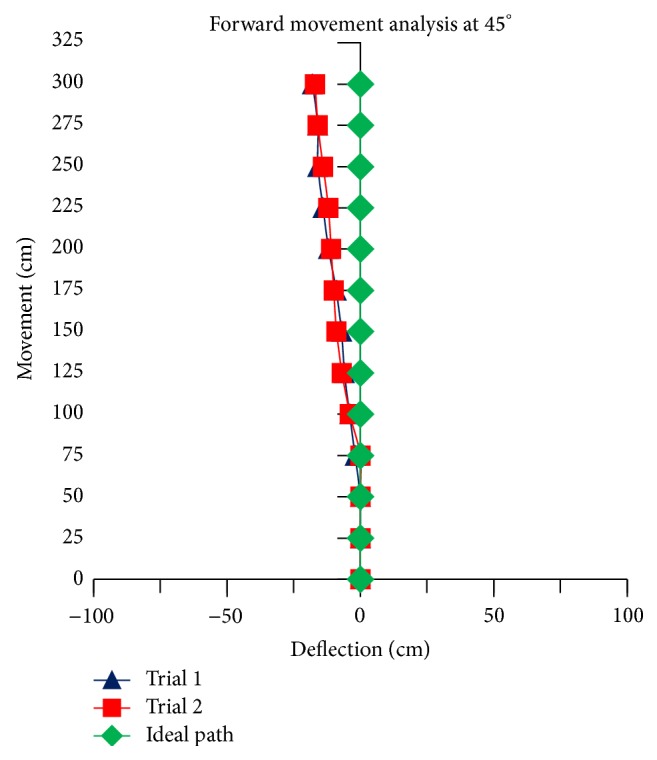
Forward movement versus deflections at *θ* = 45°.

**Figure 13:**
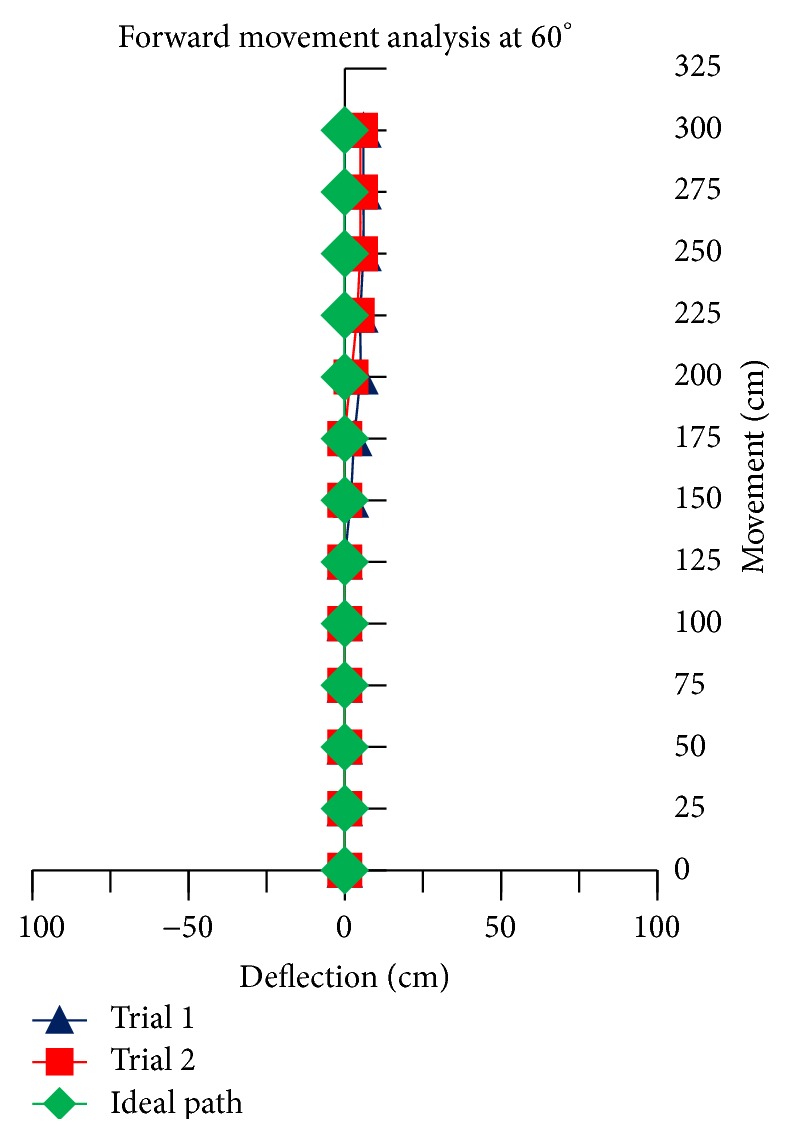
Forward movement versus deflections at *θ* = 60°.

**Figure 14:**
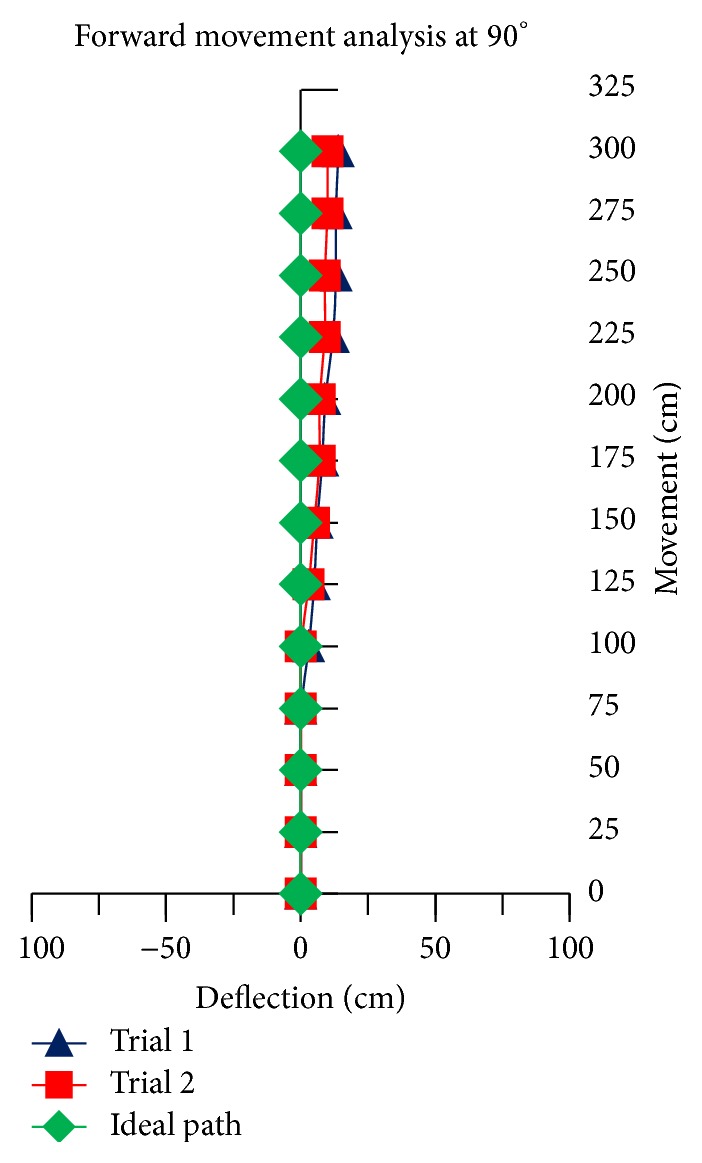
Forward movement versus deflections at *θ* = 90°.

**Figure 15:**
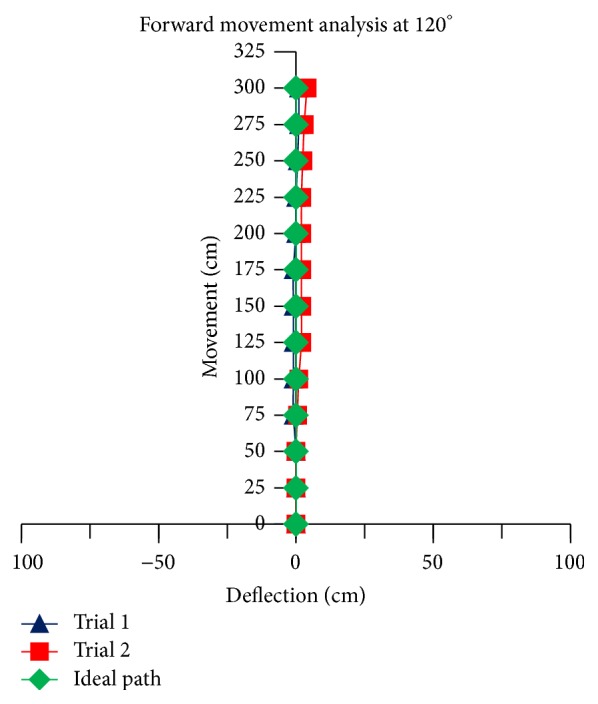
Forward movement versus deflections at *θ* = 120°.

The forward movement analysis shows that the deflections are minimum at angles *θ* = 120° and 60°, which are almost negligible.  Figure 10 shows one of the real-time experiments of the forward movement analysis of TWOMR at *θ* = 45°. It was moved up to 3 metres and the deflections from the reference line were marked and analysed.

### 5.2. Reverse Movement Analysis of TWOMR

For the reverse movement analysis of TWOMR, two trials are conducted for each angle (*θ* = 30°, 45°, 60°, 90°, and 120°) and deflection readings are plotted in Figures 17, 18, 19, 20, and 21, respectively.

The reverse movement analysis also shows that the deflections are minimum at angles *θ* = 120° and 60°, which are almost negligible.

**Figure 16:**
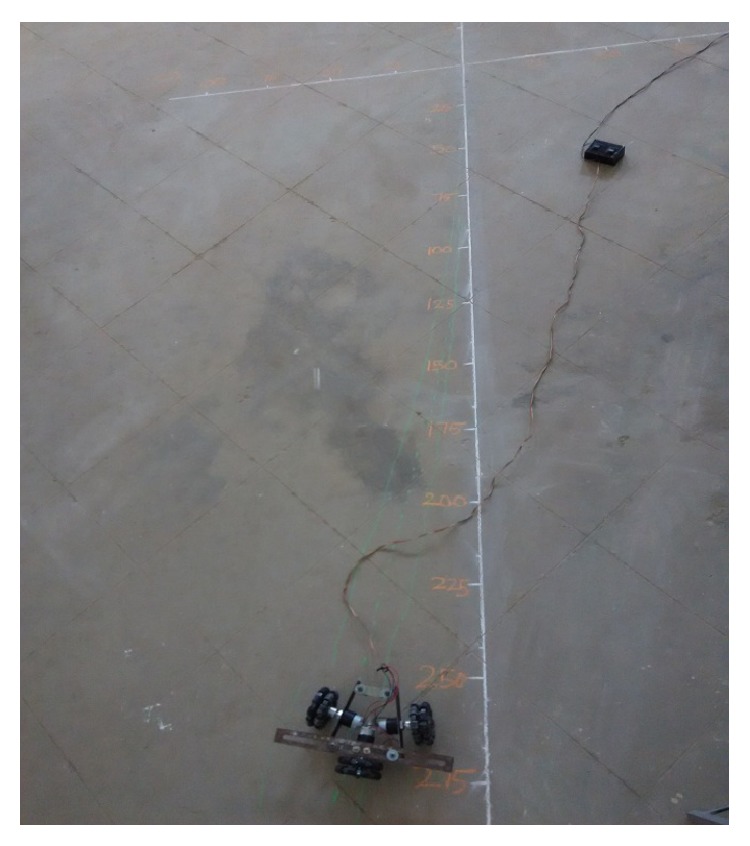
Real-time experiment of reverse movement analysis of TWOMR at *θ* = 45°.

**Figure 17:**
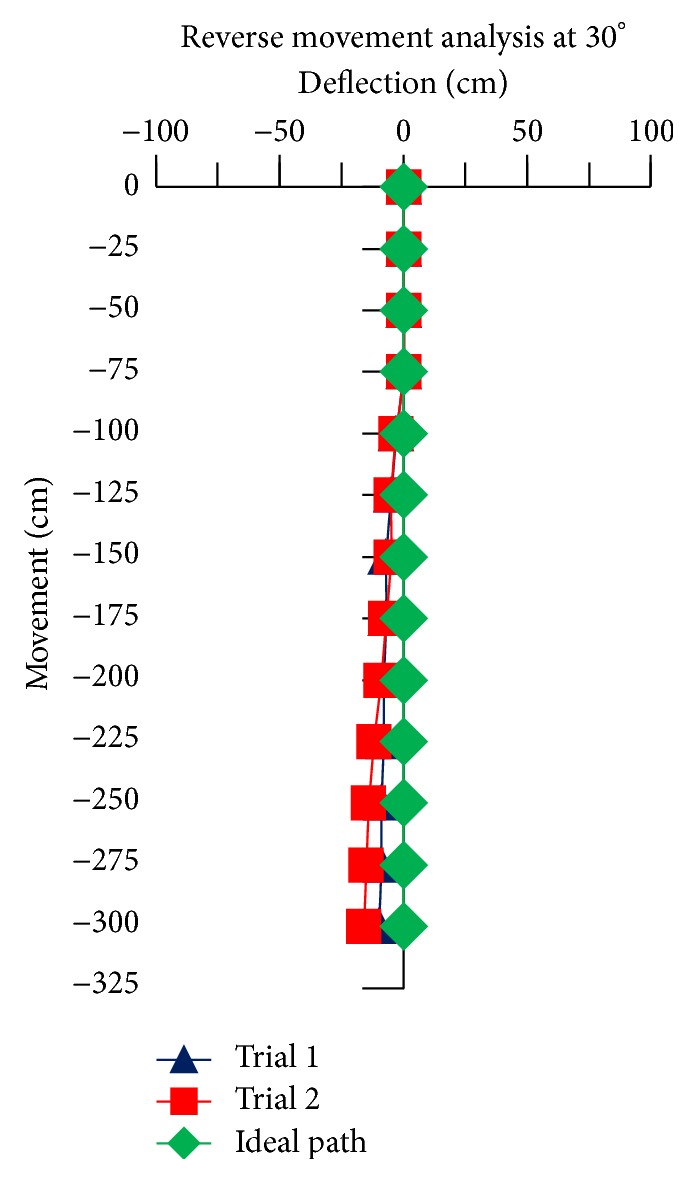
Reverse movement versus deflections at *θ* = 30°.

**Figure 18:**
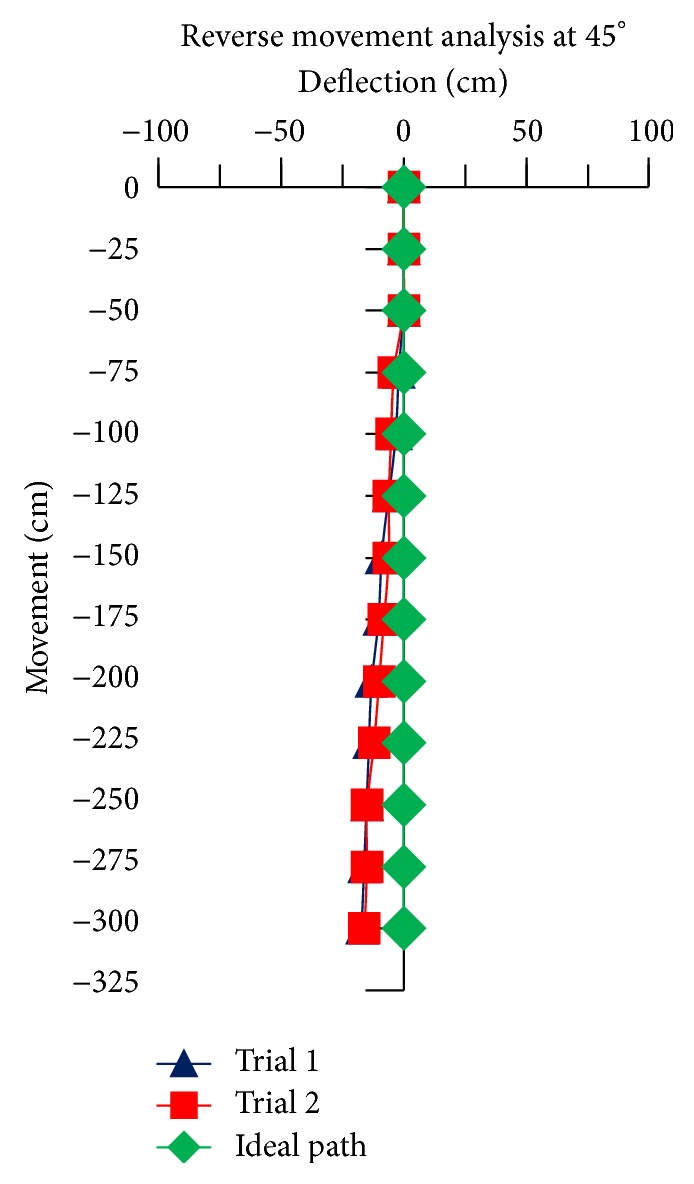
Reverse movement versus deflections at *θ* = 45°.

**Figure 19:**
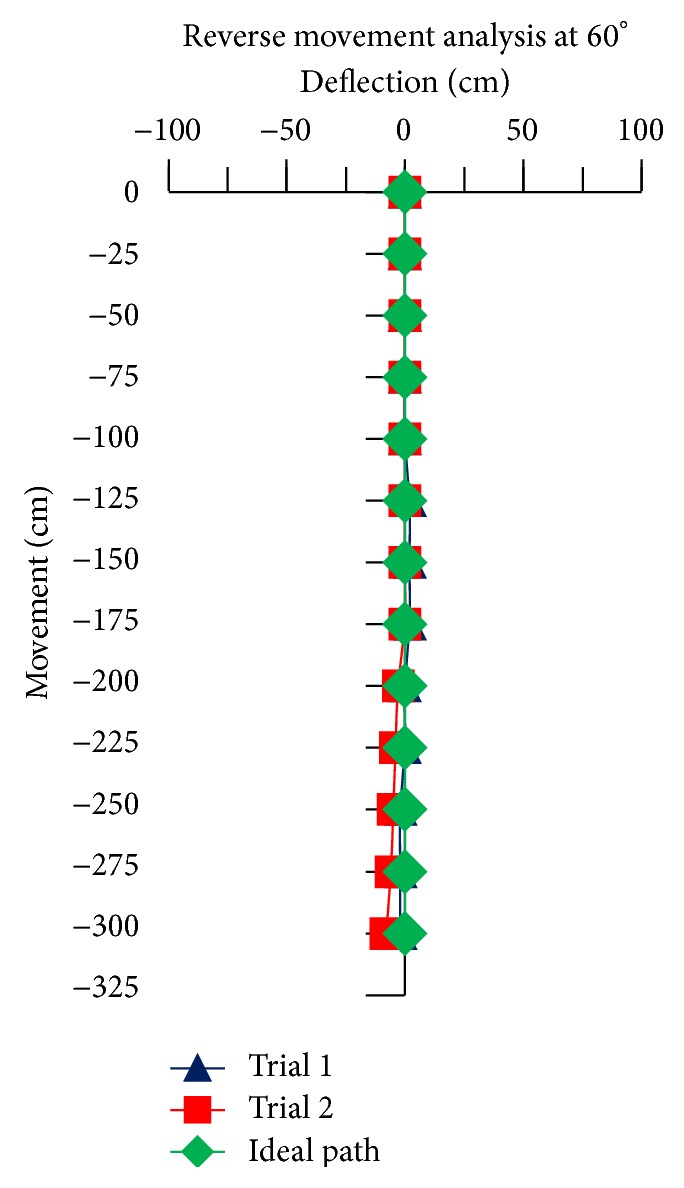
Reverse movement versus deflections at *θ* = 60°.

**Figure 20:**
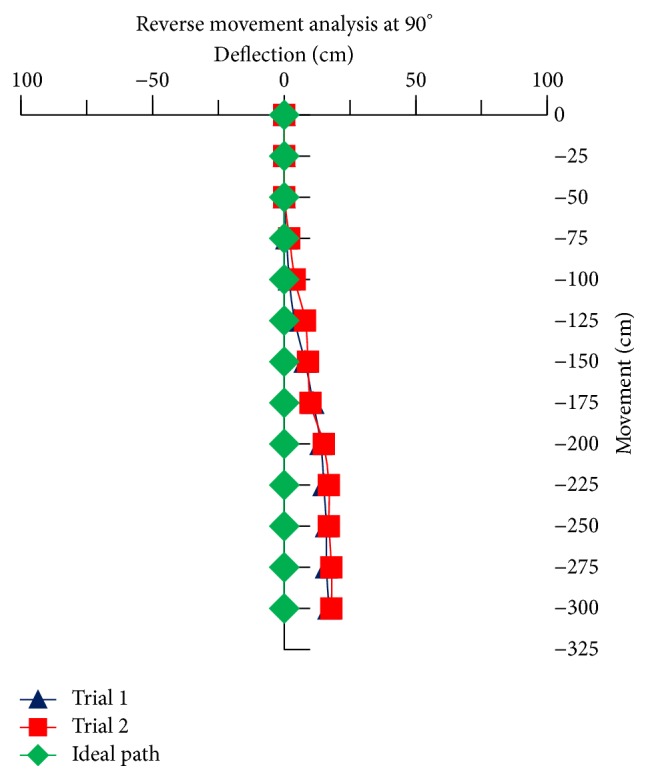
Reverse movement versus deflections at *θ* = 90°.

**Figure 21:**
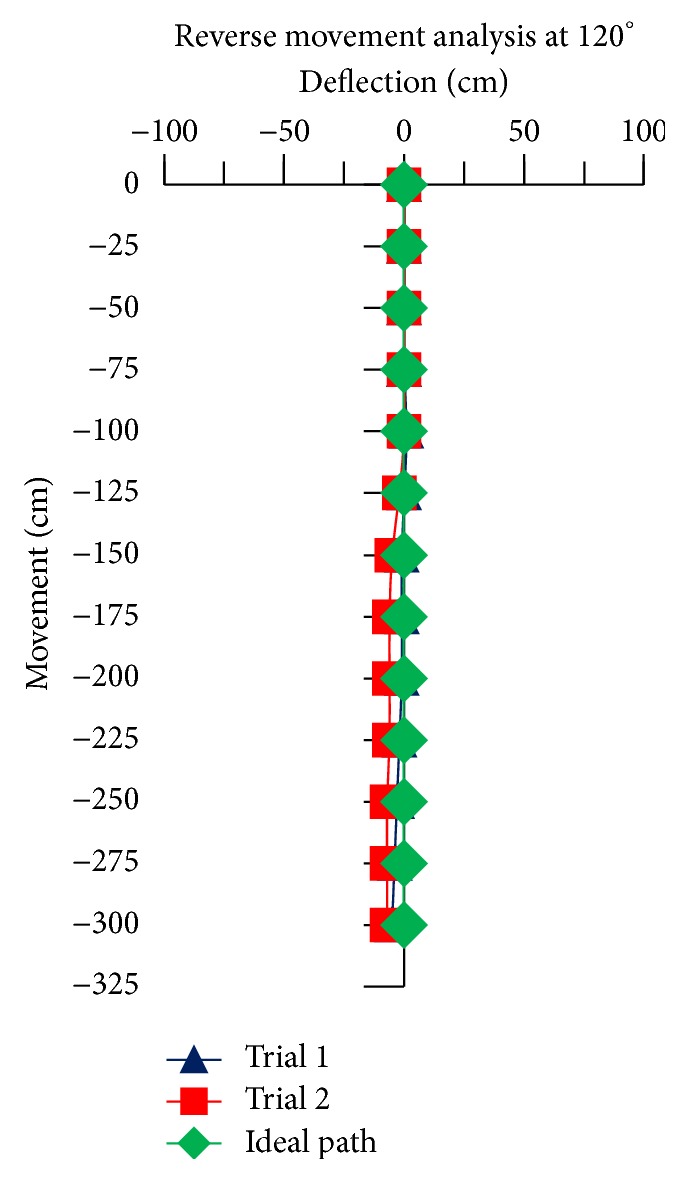
Reverse movement versus deflections at *θ* = 120°.

Hence, the deflections are arising at almost every angle (*θ*). And these deflections are minimum and almost negligible at angles *θ* = 120° and 60°, in both forward and reverse direction. Hence, these two angles are optimum angles for the forward and reverse movement of TWOMR and its performance is also very efficient. One of the real-time experiments of the reverse movement analysis of TWOMR at *θ* = 45° is shown in Figure 16. It was moved up to 3 metres and the deflections from the reference line were marked and analysed.

## 6. Discussions and Conclusions

A practical implementation of an omnidirectional mobile robot was the main focus of this work. A unique device of Angle Variable Chassis (AVC) was designed, and a three-wheeled omnidirectional mobile robot (TWOMR) was implemented and its kinematics model was described. A prototype of TWOMR was practically run on the cement floor and several experimental results indicated that the omnidirectional mobile robot had a desirable full mobility and smooth motion.

There are some errors originated from several sources such as the nonsteady condition in omnidirectional wheels during motion, the effect of friction in working model, and the effect of omniwheel rollers on omniwheel. Irrespective of these errors, TWOMR showed effective movement at angles 120° and 60°. Hence, these two angles are optimum angles for the movement of TWOMR, as the deflections are minimum and negligible. The TWOMR has greater speed at 60° compared to 120°. Hence, 60° is most preferred for the works to be done quickly. These TWOMRs are suitable and applicable in congested environments and radiation environments where humans cannot work.

Omnidirectional mobile robot can move in all directions without steering the wheels and it can rotate about its axis. In this paper, for experimental purpose, only the linear movement analysis has been done. However, there are minimum and negligible deflections arising. Hence, the control of the deflections, movement analysis on various surfaces, nonlinear movement analysis, and the ideal movement of the TWOMR will be done in future.

## Figures and Tables

**Table 1:** Specifications of omniwheel.

Wheel	Specifications
Outer diameter	100 mm
Bore (hole) diameter	13 mm with 3 mm key
Wheel thickness	38 mm
Number of rollers	2 rows and 9 rollers each
Weight	30 g
Load capacity	20 kg

**Table 2:** Specifications of DC motor.

Motor	Specifications
Speed	45 rpm
Torque	0.1961 Nm
Voltage	12 V

**Table 3:** Relation between angles *θ* and *δ*.

S. number	Angle (*θ*)	Angle (*δ*)	Resultant velocity
1	30°	75°	±2 sin⁡(75°)*ẏ*
2	45°	67.5°	±2 sin⁡(67.5°)*ẏ*
3	60°	60°	±2 sin⁡(60°)*ẏ*
4	90°	45°	±2 sin⁡(45°)*ẏ*
5	120°	30°	±2 sin⁡(30°)*ẏ*

The positive sign corresponds to the forward velocity *V*_*f*_ and the negative sign to the reverse velocity *V*_*r*_.

## References

[1] Lee Y.-C., Lee D. V., Chung J. H., Bennett D. A., Velinsky S. A. (2006). Design and control of the ball wheel drive mechanism for a robust omnidirectional wheeled mobile platform. *Romansy 16*.

[2] Doroftei I., Grosu V., Spinu V. (2008). Design and Control of an Omni-Directional Mobile Robot. *Novel Algorithms and Techniques in Telecommunications, Automation and Industrial Electronics*.

[3] Guorong Y., Haibing Z. (2001). A new kind of wheel-model all-directional moving mechanism. *Journal of Harbin Institute of Technology*.

[4] Chao Z. (2007). *Research on the design and control of a robot Omni-directional platform [M.S. thesis]*.

[5] Hirata Y., Koike Y., Liu Z., Kosuge K. Development of omni-directional mobile base with servo brakes for passive dance partner robot.

[6] Endo M., Hirose K., Sugahara Y. (2009). Trajectory generation for multiple robots of a car transportation system. *Distributed Autonomous Robotic Systems 8*.

[7] Ye C., Ma S., Hui L. (2011). An omnidirectional mobile robot. *Science China Information Sciences*.

[8] Kim S., Jeong I., Lee S. (2007). Systematic isotropy analysis of a mobile robot with three active caster wheels. *Advanced Intelligent Computing Theories and Applications. With Aspects of Theoretical and Methodological Issues: Third International Conference on Intelligent Computing, ICIC 2007 Qingdao, China, August 21–24, 2007 Proceedings*.

[9] Huang S.-B., Chen D.-S., Gong H.-Q. (2012). Research on the impact of the number of wheels and layouts to the performances of Omni-directional chassis. *Intelligent Robotics and Applications: 5th International Conference, ICIRA 2012, Montreal, QC, Canada, October 3–5, 2012, Proceedings, Part III*.

[10] Jung E.-J., Yi B.-J., Kim W. K. (2011). Motion planning algorithms of an omni-directional mobile robot with active caster wheels. *Intelligent Service Robotics*.

[11] Zou J.-T., Chiang F.-C., Su K. L. (2011). The study of path error for an omnidirectional home care mobile robot. *Artificial Life and Robotics*.

[12] Asama H., Sato M., Bogoni L., Kaetsu H., Matsumoto A., Endo I. Development of an omni-directional mobile robot with 3 DOF decoupling drive mechanism.

[13] Yi B.-J., Kim W. K. (2002). The kinematics for redundantly actuated omnidirectional mobile robots. *Journal of Robotic Systems*.

[14] Wada M., Takagi A., Mori S. Caster drive mechanisms for holonomic and omnidirectional mobile platforms with no over constraint.

[15] Ushimi N., Yamamoto M., Mohri A. Two wheels caster type odometer for omni-directional vehicles.

[16] Saha S. K., Angeles J., Darcovich J. (1995). The design of kinematically isotropic rolling robots with omnidirectional wheels. *Mechanism and Machine Theory*.

[17] Batlle J. A., Barjau A. (2009). Holonomy in mobile robots. *Robotics and Autonomous Systems*.

[18] Mohanraj A. P., Elango A., Ragavendhiran D., Raja P. V., Ashok K. (2014). Design and movement analysis of single roller Omni directional wheeled robot for different assembly structures. *Applied Mechanics and Materials*.

[19] Salih J. E. M., Rizon M., Yaacob S. (2006). Designing omni-directional mobile robot with mecanum wheel. *American Journal of Applied Sciences*.

[20] Leow Y. P., Low K. H., Loh W. K. Kinematic modelling and analysis of mobile robots with omni-directional wheels.

